# Disarming *Campylobacter hepaticus* – the *in vitro* and *in vivo* efficacy of natural antimicrobials as anti-virulence proxies

**DOI:** 10.1016/j.psj.2025.106160

**Published:** 2025-11-26

**Authors:** Ana-Maria Imbrea, Igori Balta, Sorin Morariu, Lavinia Stef, Ioan Pet, Claudia Loredana Crista, Ducu Stef, Florica Morariu, Nicolae Corcionivoschi

**Affiliations:** a Faculty of Bioengineering of Animal Resources, University of Life Sciences King Mihai I from Timisoara, 300645 Timisoara, Romania; b Faculty of Veterinary Medicine, University of Life Sciences King Mihai I from Timisoara, 300645 Timisoara, Romania; c Faculty of Food Engineering, University of Life Sciences King Mihai I from Timisoara, Timisoara, 300645, Romania; d Bacteriology Branch, Veterinary Sciences Division, Agri-Food and Biosciences Institute, Belfast BT4 3SD, Northern Ireland, UK; g Academy of Romanian Scientists, Ilfov Street, No. 3, 050044 Bucharest, Romania

**Keywords:** *Campylobacter hepaticus*, Natural antimicrobials, Virulence, Barrier integrity, Oxidative stress

## Abstract

*Campylobacter hepaticus* is responsible for the emergence of Spotty Liver Disease (SLD) in commercial layer hens. Our experimental *in vitro* approach demonstrates that natural antimicrobial mixtures (AuraShield - As) can significantly inhibit (*P* < 0.0001) the growth and motility of *C. hepaticus*. The antimicrobial acted as a chemoattractant for the pathogen, an effect which is concentration-dependent and leads to reduced bacterial motility. The As reduced *C. hepaticus* invasion of chicken liver cells (LMH cells) by strengthening TEER. The As also modulates inflammatory responses (notably by lowering IL-8 expression via the NF-κB pathway) and enhances host cell defences. We demonstrate that the presence of As (0.5%) during the infection of LMH cells by *C. hepaticus* significantly mitigates oxidative stress by reducing the detectable levels of H_2_O_2_ released into the culture supernatants (*P*<0.0001). We also show that a reduced H_2_O_2_ level will not activate the NF-kB pathway, resulting in low levels of IL-8 orthologs expression. *In vivo*, As was able to significantly reduce (*P*=0.03) not only the number of *C. hepaticus* identified in the livers of the infected hens but also the number of liver lesions, while restoring the integrity of the intestinal barrier. These *in vitro* and *in vivo* findings present new opportunities for enhanced SLD management strategies that integrate dietary innovations with improved flock biosecurity. By adopting a holistic approach that combines dietary, environmental, and management interventions, producers may achieve more sustainable and effective outcomes in the ongoing battle against *Campylobacter hepaticus* and Spotty Liver Disease.

## Introduction

Since the mid-2010s, *Campylobacter hepaticus* has emerged as a recent bacterial pathogen causing Spotty Liver Disease (SLD), also known as avian vibrionic hepatitis (AVH), in poultry. This disease is increasingly concerning in commercial layer hens (Ienes-Lima et al., 2023). Initially described as a “vibrio-like” organism in the 1950s, SLD largely disappeared during the cage-housing era but has re-emerged worldwide with the shift towards free-range and organic production systems (Courtice et al., 2018; Ienes-Lima et al., 2023). However, the precise identification of the thermophilic *C. hepaticus* was only formally established in 2015-2016 (Phung et al., 2020). The pathogen is fastidious, bile-tolerant, and, like other Campylobacters, is capable of entering a viable-but-non-culturable (VBNC) state, which is slower-growing than *C. jejuni and C. coli*, usually complicating culturing and field diagnostics, creating confounding “clearance” claims if based on culture methods (Phung et al., 2020).

In affected flocks, *C. hepaticus* infection leads to multifocal necrotic hepatitis usually visible as grey-white spots on the liver and systemic illness, often with no previously developed overt signs, resulting in significant drops in egg production and increased mortality, making SLD a serious economic threat to the egg industry, while also incurring costs for veterinary care, biosecurity upgrades, and management changes (Van et al., 2022; Wade et al., 2024). Interestingly, until recently, *C. hepaticus* was recognised as the sole causative agent of SLD; however, *Campylobacter bilis* has now been identified as a second novel species that causes SLD (Van et al., 2023). Nevertheless, *C. hepaticus* remains the primary etiologic agent of SLD in poultry worldwide. Outbreaks can cause up to 10% flock mortality and a decline in egg production *of* ∼25% in layers (Van et al., 2023). Notably, SLD has been reported across both Americas, Europe, Asia, and Australia, especially in cage-free systems where outdoor exposure and environmental reservoirs facilitate the spread of *C. hepaticus* (Ienes-Lima et al., 2023). For instance, in Australia, researchers observed a sharp rise in SLD incidence over the last decade, and it is now considered one of the most significant health challenges in Australian free-range egg production (Van et al., 2019). In the United States, SLD has been confirmed in multiple states (i.e., Iowa, Florida, Georgia, and others) as producers move toward cage-free housing (Ienes-Lima et al., 2023). Europe and the UK have also documented SLD outbreaks after periods of dormancy. The seasonality of SLD has been noted anecdotally, with more outbreaks occurring in warmer months, possibly due to better survival of *Campylobacter* in the environment or heat stress in birds (Ienes-Lima et al., 2023).

Currently, there are no approved treatments or commercial vaccines for the disease, and management has relied mainly on antimicrobial interventions and enhanced biosecurity measures (Becerra et al., 2025; Wade et al., 2024). A notable example of the increased efficacy of mixed antimicrobials was the standardised blend of isoquinoline alkaloids (plant-derived compounds), such as sanguinarine and chelerythrine, from *Macleaya* or *Chelidonium* spp. (Quinteros et al., 2021). Feeding laying hens a low inclusion of these alkaloids (200 mg/kg) resulted in a partial protective effect with fewer and milder liver lesions after *C. hepaticus* exposure compared to controls (Quinteros et al., 2021). Other improvements were associated with increased weight gain in hens, higher feed consumption, and increased egg production, while also significantly reducing the expression of proinflammatory cytokines (IL-8) enabled by the infection. Their study suggested that isoquinoline phytobiotics can mitigate the impact of SLD, presumably by both direct antimicrobial action in the gut/liver and by modulating host responses due to the anti-inflammatory properties of isoquinoline alkaloids, previously demonstrated in other studies (Danielewski et al., 2025; Meng et al., 2024; Zhang et al., 2020). Mechanistically, as an antimicrobial, sanguinarine and its analogues potentiate antibiotic action by intercalating into DNA and binding to enzymes, particularly inhibiting bacterial cell division (Beuria et al., 2005; Lu et al., 2022). Sanguinarine has been shown to bind the FtsZ protein, preventing it from forming the Z-ring needed for cytokinesis (Beuria et al., 2005; Jubair et al., 2021). By blocking FtsZ polymerisation, sanguinarine essentially causes the bacteria to fail in dividing, resulting in filamentation or cell death. In *Campylobacter*, which also relies on FtsZ for binary fission, a similar phenomenon would occur, thereby possibly reducing bacterial multiplication (Tripathy & Sahu, 2019).

A recent trial evaluated the potential effects of water acidification treatments (e.g., oregano, apple cider vinegar, and citric acid) on controlling *C. hepaticus* (Becerra et al., 2025). The results showed that the supplementation with oregano, apple cider vinegar, or citric acid was insufficient to eliminate *C. hepaticus* in experimentally challenged birds, as over ≈45% of infected birds remained PCR-positive in liver samples following treatment. Moreover, horizontal transmission was evident, with more than ≈30% of naïve, non-challenged birds testing positive after co-housing with challenged birds (Becerra et al., 2025). In conclusion, the acidification of drinking water showed potential in mitigating bacterial spread, since *C. hepaticus* could not be cultured from bile in naïve birds exposed to treated groups. The authors suggested a possible role in potentially reducing bacterial load and transmission or shedding dynamics.

Understanding the biological mechanisms by which natural antimicrobials can disarm pathogens is vital, as it offers a novel strategic intervention to reduce their virulence, unlike antibiotics. We have previously shown that antimicrobials in mixture (AuraShield), are capable of reducing viral (Balta et al., 2020) and bacterial infections (Balta et al., 2024). Therefore, this paper aimed to investigate the role of natural antimicrobials in mixture (As) in preventing, *in vitro*, the infection of chicken liver epithelial cells (LMH cells) by *C. hepaticus*. We have also assessed its ability to reduce *C. hepaticus* infection and disease onset in hens, because their effectiveness can vary depending on factors such as flock management, environmental conditions, and the specific strains of *C. hepaticus* involved. Future research is essential to elucidate further the mechanisms by which these interventions act and to optimise their application in real-world settings.

## Material and methods

### Bacterial growth and antimicrobial mixture

*C. hepaticus* NCTC 13823^T^ was cultured on Brucella agar (BB) with 5% horse blood and incubated at 37°C in microaerobic conditions (85% N_2_, 5% O_2_, and 10% CO_2_) in a Don Whitley MACS-VA500 microaerophilic workstation (Davidson & Hardy Ltd., United Kingdom). The natural antimicrobial mixture, AuraShield (As), contains 5% maltodextrin, 1% sodium chloride, 42% citric acid, 18% sodium citrate, 10% silica, 12% malic acid, 9% citrus extract, and 3% olive extract (w/w). Bio-Science Nutrition Ireland supplied the raw materials. Experiments were carried out in triplicate (*n* = 3).

### Minimum inhibitory (MIC) and minimum bactericidal (MBC) concentration against *C. hepaticus*

Both the MIC and the MBC were determined as previously described (Balta, Linton, Pinkerton, Kelly, Stef, et al., 2021). The two-fold tube dilution method was used to determine the lowest concentration that inhibited bacterial growth (MIC), and the lowest concentration that induced bacterial death (MBC). AuraShield was diluted (8–0.015625% v/v) in BB (Brucella Broth) broth and vortexed. Bacterial cultures were collected by centrifugation, washed in PBS (2X), and resuspended in BB at 1 × 10^6^ CFU/mL. Non-inoculated 5 mL tubes containing BB were used as negative controls, whilst BB tubes without AuraShield were inoculated with individual bacterial cultures as positive controls. The bacteria-containing tubes were incubated at 37°C for 48h. Tubes that did not show visible growth were above the MIC. Then, 100 μL was taken from each tube for inoculation and incubated at 37°C for 24 h on BB agar. The highest dilution of each antimicrobial with no microbial growth was considered the MBC. To determine the sub-inhibitory concentrations used, *C. hepaticus* was exposed to different concentrations of the antimicrobial mixture. The highest concentrations of antimicrobial that showed no effect on survivability and no growth inhibition (same growth kinetics as the control) were used in subsequent experiments.

### Invasion assay of LMH cells

The invasiveness of the *C. hepaticus* NCTC 13823^T^ strain to LMH (ATCC CRL-2117) chicken hepatocellular carcinoma epithelial cells was performed using the gentamycin protection assay as previously described (Van, Elshagmani, et al., 2017). Briefly, the LMH cells (ATCC 2117) were grown in flasks coated with 0.1% gelatine in Waymouth’s medium (Gibco, UK) supplemented with 10% foetal bovine serum at 37°C in a 5% CO_2_ and transferred to 24-well plates and seeded at 10^5^ cells/well and cultured for 48h before infection. The *C. hepaticus* strain was diluted in broth to an optical density at 600 nm (OD_600_) of 0.1, corresponding to approximately 10^8^ CFU/mL (10 MOI). A 100 μL volume of this bacterial suspension was then used to inoculate the LMH cells. The viable counts of the bacterial suspensions used in the assay were directly measured by plating out dilutions on Brucella agar plates. The 24-well plates were incubated at 37°C under microaerobic conditions for 5 h, allowing the bacterial strains to invade the LMH cells. Cells were washed twice with PBS, then 1mL of Waymouth’s medium/FBS containing 400 μg/mL gentamycin was added to each well. The plates were incubated in 5% CO_2_ at 37°C for 90 minutes to kill bacterial cells that had not invaded. The cells were then lysed by adding 200 μL of 0.3% Triton X-100 to each well. The cell lysates were diluted by the addition of 800 μL of PBS, and the plates were incubated in 5% CO_2_ at 37°C for 15 minutes. The lysate was further diluted and plated onto Brucella agar plates to evaluate the number of viable bacteria that had invaded the LMH cells. The invasive ability was expressed as the percentage of the inoculum surviving the gentamycin treatment relative to the initial inoculum. The invasion assay was performed at least twice, in triplicate at each time point. For statistical analysis of the data, the mean values were compared using ANOVA and Dunnett’s test. Mean values were considered to be significantly different if the *P* value was less than 0.01. Cell survival was evaluated by adding 10 μL of the MTT reagent (0.5 mg /mL MTT) to each well and incubating for an additional 3 h. This medium was then removed, and 100 μL of the solubilization solution was added to dissolve the MTT formazan. The plate was incubated overnight at 37°C with 5% CO2. The absorbance of the MTT purple colour was measured on a multiwell plate reader (FLUOstar Omega, BMG Labtech, UK) using a 570 nm filter. Cell viability was expressed as a percentage of the control.

### Transepithelial electrical resistance (TEER) measurements

LMH cells were plated onto transwells (5 × 10^4^; 6.5 mm diameter; 0.4 μm—pore size; Corning) and grown until apical junctional complexes developed. Transwells were infected apically with *C. hepaticus*. TEER was measured 48 h after infection using an EVOM X meter connected to an Endohm chamber (World Precision Instruments). The presented results are representative of at least three independent experiments.

### Measurement of cellular H_2_O_2_ release

Hydrogen peroxide (H_2_O_2_) production was measured using a Hydrogen Peroxide Detection Kit (Enzo Life Sciences) or Amplex® UltraRed /HRP (Thermo Fischer Scientific, UK) according to the manufacturer’s instructions. Briefly, the lysis buffer or culture media (50 mL) was mixed with the Amplex® UltraRed /HRP (Thermo Fisher Scientific, UK) reagent and horseradish peroxidase, resulting in a red fluorescent oxidation product. Fluorescence was determined at 530 nm excitation and 590 nm emission using a fluorescence microplate reader (FLUOstar Omega, BMG Labtech). The concentrations of H_2_O_2_ were calculated using standard curves.

### RT PCR of IL-8 orthologs in LMH cells and quantitative enumeration of *C. hepaticus* in liver samples

Expression of orthologous genes involved in IL-8 production in infected LMH cells was performed as previously described (Larson et al., 2008), with minor modifications. Gene names and primers used are presented in Table 1. Briefly at 48h post-infection (as described above), the infected monolayers were snap-frozen in liquid nitrogen until use. RNA was isolated using RNeasy Plus Mini Kit (Qiagen, United Kingdom). The RNA was reverse-transcribed using the Transcriptor First Strand cDNA Synthesis Kit (Roche) according to the manufacturer’s protocol. The mRNA levels were determined by quantitative RT-PCR using QuantiNovaSYBR Green PCR Kit (Qiagen, United Kingdom) on a LightCycler 96 (Roche).

**Table 1:** Primers used in this study.

Gene	Primers (5′-3′)
*chCXCLi2* (*CAF*)	F: ATGAACGGCAAGCTTGGAGCT R: GCCATAAGTGCCTTTACGATCAG
*chCXCLi1* (*K60*)	F: TGGCTCTTCTCCTGATCTCAATG R: GCACTGGCATCGGAGTTCA
β actin (chicken)	F: CATCACCATTGGCAATGAGAGG R: GATTCATCGTACTCCTGCTTG C
*C. hepaticus*	G2F3: CAGGAGTTTTACCACAATTC G2R2: CAAGCTAAAACAGGTTTGG
Claudin-1	F: TGGAGGATGACCAGGTGAAGA R: CGAGCCACTCTGTTGCCATA
Claudin-3	F: CTTCATCGGCAACAACATC R: CATGGAGTCGTACACCTTG
Claudin-4	F: GCATCGCCCTGTCCGTCATC R: ACGATGTTGTTGCCCACGAAGG
Occludin	F: TCATCGCCTCCATCGTCTAC R: TCTTACTGCGCGTCTTCTGG
E-cadherin	F: GACAGGGACATGAGGCAGAA R: GCCGTGACAATGCCATTCTC
ZO-1	F: TGTAGCCACAGCAAGAGGTG R: CTGGAATGGCTCCTTGTGGT
ZO-2	F: CGGCAGCTATCAGACCACTC R: CACAGACCAGCAAGCCTACAG
ZO-3	F: CAAAGCAAGCCGGACATTTAC R: GTCAAAATGCGTCCGGATGTA
Mucin-2	F: CAGCACCAACTTCTCAGTTC R: TCTGCAGCCACACATTCTTT

The RT-PCR conditions were as follows: 2min at 50°C, 15min at 95°C followed by 40 repeats of 15s at 94°C, 30s at 59°C, and 30s at 72°C. The chicken β-actin was used as a reference gene. These data were analysed using the comparative Ct method, and the results are presented as the fold increase of gene expression of the bacteria-inoculated samples relative to the uninoculated sample. DNA preparation and quantitation of *C. hepaticus* in liver samples after necropsy were performed using qPCR as previously described (Van, Gor, et al., 2017) in triplicate. Primers used are presented in Table 1. DNA derived from *C. hepaticus* NCTC 13823^T^ was used to generate a standard curve for efficiency determination.

### RNA extraction and qRT PCR Analysis of intestinal barrier-related genes

Quantification of the relative mRNA expression levels of intestinal barrier-related genes was performed as previously described with primers included in Table 1 (Yu et al., 2023). Briefly, RNA from ileum tissue was extracted using the RNeasy Plus Mini Kit (Qiagen, United Kingdom). The RNA was reverse transcribed using Transcriptor First Strand cDNA Synthesis Kit (Roche) according to the manufacturer’s protocol. The mRNA levels were determined by quantitative RT-PCR using QuantiNova SYBR Green PCR Kit (Qiagen, United Kingdom) on a LightCycler 96 (Roche). The qPCR conditions included an initial heating step to 95°C for 1 min, followed by 40 cycles of 95°C for 20 s and 60°C for 1 min. Relative gene expression levels were evaluated using the 2 ^ΔΔCT^ method with β-actin.

### Enzyme-linked immunosorbent assay (ELISA)

Serum samples were collected at 4 weeks post-infection to monitor *C. hepaticus* IgY antibodies in infected birds. The samples were analysed as previously (Muralidharan et al., 2020). The first step consisted of Coating plate wells with *C. hepaticus* total protein extracts as the antigen and measuring the *C. hepaticus*-specific antibodies. Non-specific binding was blocked with 5% skim milk in phosphate-buffered saline (PBS) + 0.05% Tween 20 (PBST). Secondly, the primary chicken sera were diluted to 1:1000 followed by probing with goat anti-chicken secondary antibody (1:2000). The HRP Chromogenic Substrate (Thermo Fisher Scientific, UK) was added, and absorbance was measured on a microplate reader (FLUOstar Omega, BMG Labtech) at 652nm. All measurements were done in triplicate. The positive controls used in the assay were sera from birds with known anti-*C. hepaticus* antibodies and from uninfected birds.

### NF-κB activation assay

The level of NF-κB p65 activation was measured in the EHP-infected SGP cells after 5 h of infection, following LPS stimulation, or after infection and exposure to 0.5% Aq. The nuclear proteins were extracted using a Nuclear Extraction kit from Abcam (London, UK), and the NF-κB p65 activation in the supernatants was assessed with an NF-κB p65 Transcription Factor Assay Kit (Colourimetric) from Abcam, following the manufacturer’s instructions. NF-κB p65 activation was expressed as the ratio of absorbance at 450 nm (OD 450 nm), representing the amount of NF-κB p65 activation per 1 mg of total nuclear protein. Colourimetric changes were recorded at 550 nm using a FLUOstar Omega plate reader (Premier Scientific, Belfast, UK).

### Proteasome activity

The effect of As on the LMH cells' proteasome was assessed as previously described (Hurley et al., 2023) using the proteasome activity assay kit (Abcam ab107921) and following the manufacturer’s instructions. Briefly, the impact of As on the LMH cells proteosome infected with *C. hepaticus* was assessed at 48h as follows: in LMH cells only, in LMH cells stimulated with LPS (1µg/mL), in LMH cells exposed to 0.5% As, and in LMH cells infected with *C. hepaticus* with or without the presence of 0.5% As. Reads were performed at an excitation of 350 nm and an emission of 440 nm after 48 h using a microplate reader (FLUOstar Omega from BMG Labtech, Ortenberg, UK).

### Chemotaxis syringe capillary assay, and bacterial motility

In order to test the bacterial affinity for the antimicrobial mixture, we have used a previously published method with minor modifications (Lübke et al., 2018). Briefly, *C. hepaticus* grown on agar plates was resuspended in 1mL of PBS and washed 1X following harvesting by centrifuging at 4500g for 5min. The resulting pellet was adjusted to an optical density (OD_600_) of 0.5. A volume of 100μL of each of the following concentrations 0.5%, 1% and 2% As, was drawn through a 27 G hypodermic needle of a 1 mL Luer syringe. PBS only served as a control. The bacterial suspension (100 μL) was absorbed into a 200 μL disposable pipette, and the tip was sealed with parafilm. The pipette was then attached to the needle-syringe system. The entire system was then incubated for 1 hour at 37°C under microaerophilic conditions. The content of the needle-pipette tip was plated in 10-fold serial dilutions on BB agar plates for 48h at 37°C or 42°C under microaerophilic conditions, and the CFU were counted. The taxis toward As were calculated by the Relative Chemotaxis Ratio (RCR), which is the ratio of the number of bacteria in the syringe with As to the number in the control (PBS only) after 1h incubation. The experiment was performed in triplicate. *C. hepaticus* motility assay was performed as previously described (Ungureanu et al., 2019).

### *In vivo* infection assay

One hundred and forty-four Hy-Line Brown hens (15 weeks old) were housed in microbiologically safe isolators on wood shaving bedding. The temperature in the isolation unit was kept between 22 and 25°C and thermostatically controlled. Hens were divided into three groups (A, B and C) with three replicates/group (16 hens/replicate). At Day 0, Group A received broth only, and Group B received 1 × 10^9^ CFU/mL of *C. hepaticus* orally. Group C was also infected with 1 × 10^9^ CFU/mL of *C. hepaticus* but received 0.5% As in the drinking water for 2 weeks post-infection. All birds were necropsied 4 weeks post-challenge. Fig. 7A describes the trial design. At necropsy, SLD lesions on the liver were enumerated, and samples were collected to determine the recovery of *C. hepaticus* and anti-*C. hepaticus* IgY antibody in the serum of the infected groups. These experiments were performed in triplicate on three separate occasions. The experiments were performed according to the legislation in place (Law 471/2002 and government ordinance 437/2002). All procedures were subject to the approval of the local Animal Welfare Ethics Review Board. All procedures were carried out under the strict guidelines of the Animal (Scientific Procedures) Act 1986.

### Statistical analysis

Statistical analyses were performed using GraphPad software, version 11. In some cases, data were represented as mean ± SD. *P*-values < 0.05 were considered statistically significant following estimations using the Student’s *t*-test. One-way ANOVA, Two-way ANOVA, Dunnett’s and Bonferroni’s tests were used for grouped and multiple comparisons.

## Results

### The impact of As on the MIC, MBC and on the growth of *C. hepaticus*

**Fig. 1:**
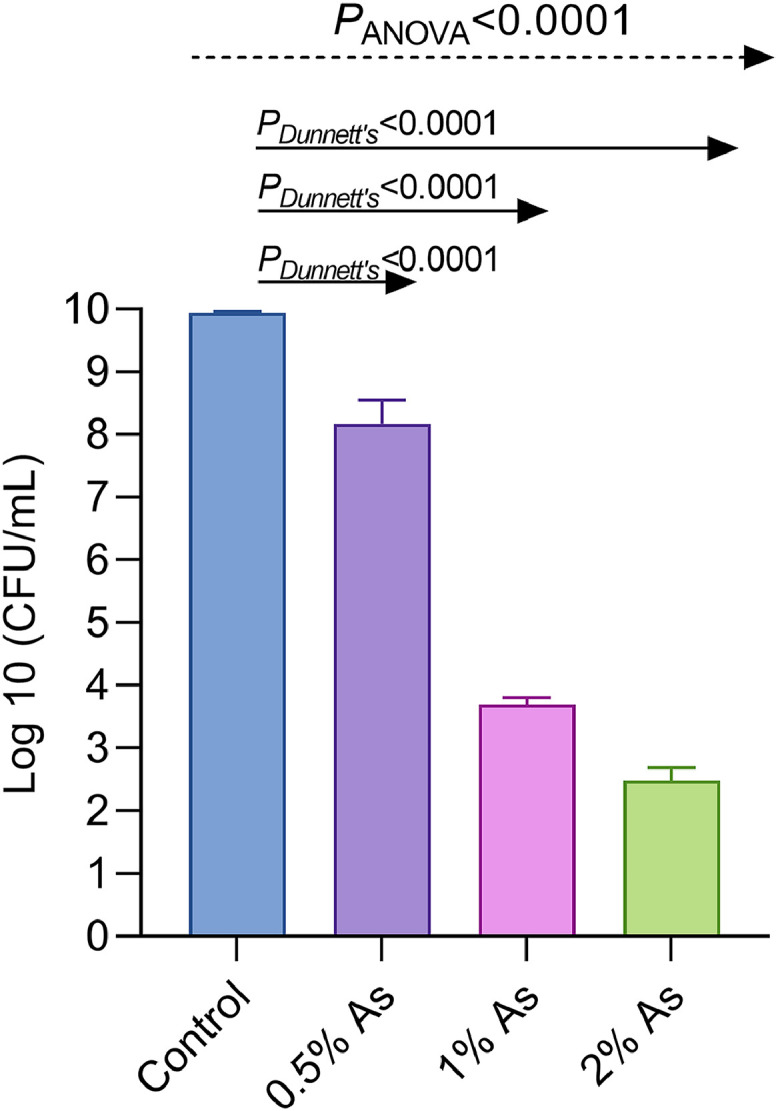
Growth of *C. hepaticus* in Brucella broth in the presence of 0.5%, 1% and 2% As. One-way ANOVA was used to analyse data for overall comparison, and Dunnett’s test was used for multiple comparisons between groups at various concentrations. Experiments were performed in triplicate and presented as log10 CFU/mL.

First, we have determined the MIC and the MBC values at which As inhibits the growth or kills *C. hepaticus*. Our results identified an MIC value of 0.5% and an MBC value of 2% As. To test the impact on bacterial growth, we performed a viable count growth curve up to 48 h to investigate the effect of 0.5%, 1%, and 2% As on *C. hepaticus* growth at the identified MIC and MBC values. This has allowed us to select the concentration of 0.5% as subinhibitory. The results show (Fig. 1) a significant effect of As on *C. hepaticus* growth (*P*<0.0001) at all three concentrations investigated as compared to the control growth test in the absence of As. The results demonstrated a dose-dependent reduction in bacterial growth with increasing As concentration, highlighting the antimicrobial efficacy of the mixture under the conditions tested.

### The affinity of *C. hepaticus* for As and the impact on bacterial motility

**Fig. 2:**
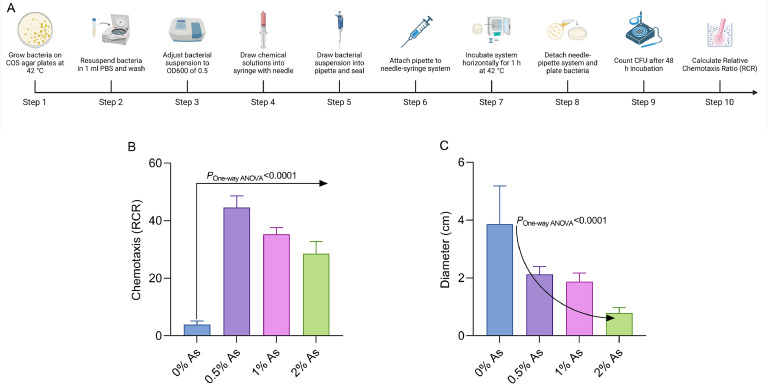
Chemotaxis and motility assay. Syringe-capillary assays were performed using 0.5%, 1%, and 2%As as the chemoattractant. Panel A describes the chemotaxis experimental design (Created with Biorender.com), and Panel B shows the number of bacteria in the syringe, as compared to the control (PBS only). The *C. hepaticus* motility assay data are presented in Panel C. Experiments were performed in triplicate, and One-way ANOVA was performed to test for significance.

The purpose of this next experiment was to help us understand whether the initial interaction between the pathogen and the antimicrobial mixture (As) is based on a survival instinct and nutritional affinity, which directly impacts its virulence. A syringe-capillary assay was used to determine the chemotactic response of *C. hepaticus* towards 0.5%, 1% and 2% As (Fig. 2A). The concentration of 0.5% As was found to have the highest chemoattractant effect towards *C. hepaticus*. As the concentration of As increases (1% and 2%), the RCR values decrease but remain significantly higher than those of the control without As (0%) (Fig. 2B). One explanation for this decrease could be that these concentrations either start killing the pathogen or disarm it by inhibiting bacterial virulence factors, such as slowing down bacterial motility. To verify the hypothesis that As has an impact on *C. hepaticus* motility, we performed a motility assay (Fig. 2C) and confirmed a significant reduction in *C. hepaticus* motility (*P* < 0.0001) after 1h of exposure to the antimicrobial mixture. These results confirm that the bacterial pathogen has an affinity for the antimicrobial mix, ultimately affecting bacterial motility.

### The *in vitro* effect of As on *C. hepaticus* virulence and epithelial barrier integrity

**Fig. 3:**
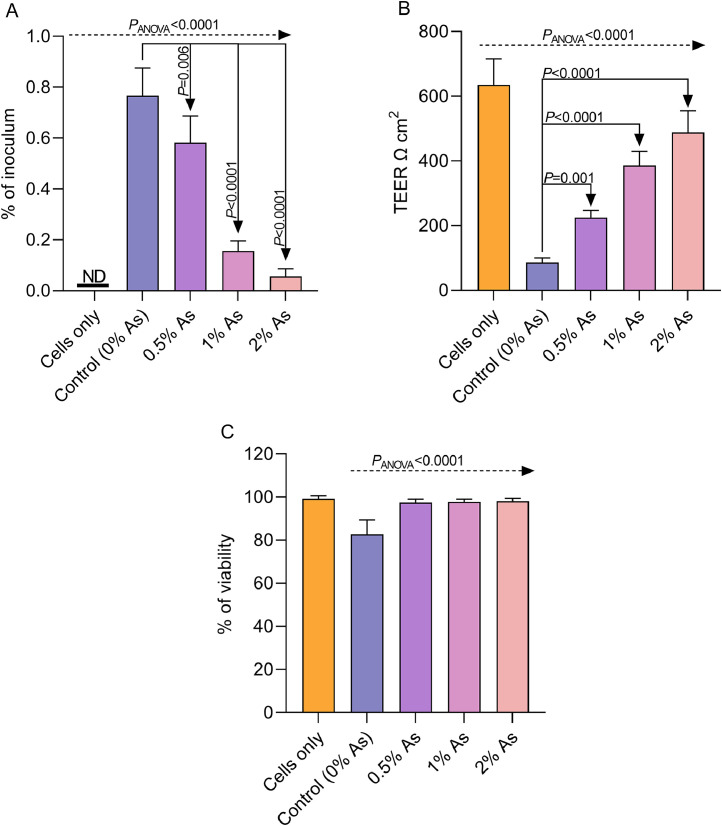
The impact of As (0.5%, 1% and 2%) on the invasion abilities of *C. hepaticus* (A), TEER (B) and cell viability (C). The invasion results are presented as percentages of internalised bacterial cells compared to the initial inoculum. One-way ANOVA and the Dunnett’s test for multiple comparisons were used to test for significance. A value of *P*<0.01 was considered significant. The means and standard deviations are shown by bars.

Next, we have tested the ability of As to reduce the invasive capabilities of the *C. hepaticus* NCTC 13823^T^ strain. The ability of the pathogen to invade LMH cells (Fig. 3A) decreased significantly (*P*<0.0001) in the presence of all three As concentrations tested (0.5%, 1% and 2%). The interaction of *C. hepaticus* with the LMH cells also led to a significant decrease in transepithelial resistance (TEER); however, considerable recovery of TEER was detected when As was present during the infection assay (Fig. 3B). Moreover, cell viability was also improved in the presence of As, suggesting that the antimicrobial mixture doesn’t have a cytotoxic effect on the host cells. These results indicate that the antimicrobial mix can reduce the *in vitro* infectivity of the *C. hepaticus* strain, an observation which is likely to be reflected *in vivo*.

### The As downregulates the expression of IL-8 orthologs (*chCXCLi1* and *chCXCLi2*) in *C. hepaticus* infected LMH cells

**Fig. 4:**
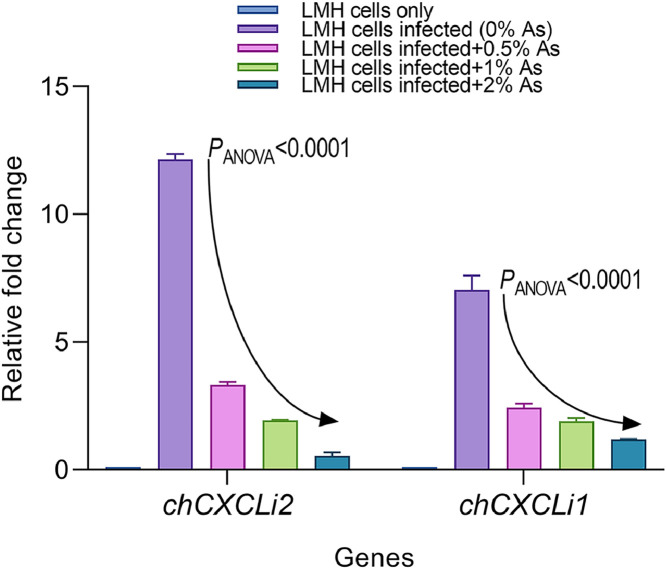
Analysis of *chCXCLi2* and *chCXCLi1* expression in LMH cells. The relative fold changes in transcript levels were determined from *C. hepaticus*-infected cells using the comparative Ct method and normalised as described in Materials and Methods. Data was analysed with the Two-way ANOVA test for overall comparison, and the Dunnett’s test for multiple comparisons between groups at various concentrations. *P*-values are shown on the graph.

To estimate the impact of As on the proinflammatory response in infected LMH cells, we measured the expression of *chCXCLi1* and *chCXCLi2* genes involved in this response. Cells only and cells infected with *C. hepaticus*, but untreated with As, were used as controls. As indicated in Fig. 4, the expression of both *chCXCLi1* and *chCXCLi2* was downregulated significantly (*P*_ANOVA_<0.0001) when compared to the levels in the infected but untreated cells. To further investigate the significance, we have also performed Dunnett’s test to account for the individual differences between the infected but untreated control and the *C. hepaticus*-infected LMH cells in the presence of 0.5%, 1%, and 2% respectively. Following Dunnett’s test analysis, significant differences were identified in all cases (*P*<0.0001). These results suggest that in the presence of As, an IL-8-based proinflammatory response is likely to be significantly reduced.

### Proteasome activity in the presence of As

**Fig. 5:**
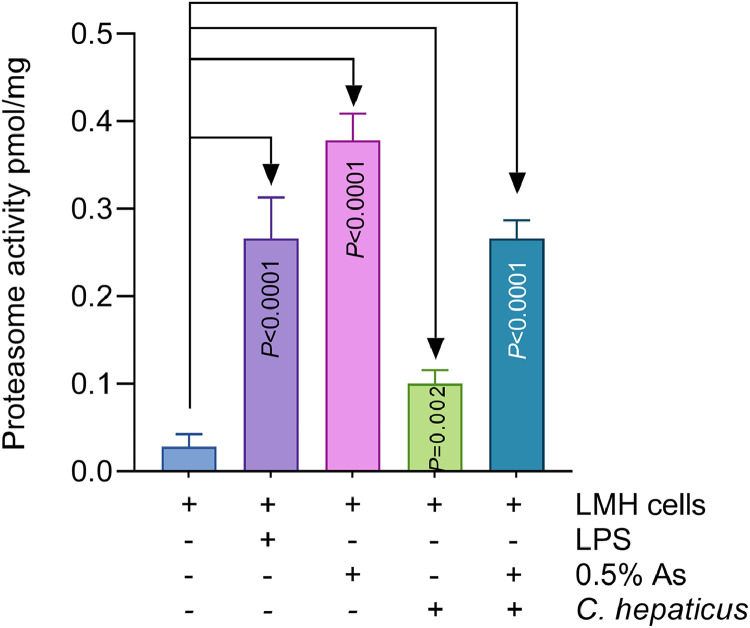
Proteasome activity in LMH cells stimulated with LPS 1 µg/mL (*P*<0.0001), with 0.5% As (*P*<0.0001), infected with *C. hepaticus* (*P*=0.002) or infected with *C. hepaticus* in the presence of 0.5% As. Dunnett’s multiple comparison test was used to analyse the data.

Following the negative impact on IL-8 expression, we next analysed the effect on the host proteasome, given the eukaryotic proteolytic system’s role as the first line of defence during infection. As indicated in Fig. 5, after 48 h, exposure of LMH cells to LPS (µg/mL), 0.5% As, or upon infection with *C. hepaticus* in the presence or absence of 0.5% As, resulted in a significant activation of proteasome activity (*P* values indicated in Fig. 5). These results suggest that the natural antimicrobial mixture can activate the host proteasome as the first line of defence during *C. hepaticus* infection of LMH cells.

### The presence of As during infection results in cellular NF-kB pathway inactivation

**Fig. 6:**
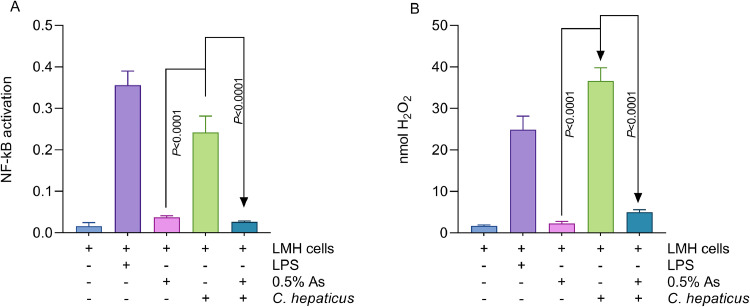
The impact of 0.5% As on the NF-kB pathway activation during infection of LMH cells by *C. hepaticus* (Panel A). Panel B represents the amount of H_2_O_2_ released in the extracellular space during infection in the presence/absence of 0.5% As. Dunnett’s test was used to analyse the data. Significant *P* values are shown on the graph.

Given that the NF-κB pathway regulates IL-8 expression, we next investigated whether As can modulate NF-κB activation during infection with *C. hepaticus* in LMH cells. *C. hepaticus* infection triggered the activation of the NF-kB pathway (Fig. 6A), which was significantly inactivated by the inclusion of 0.5% As (*P*<0.0001). Stimulation of LMH cells with LPS also activated the NF-kB pathway. We hypothesise that the NF-kB pathway is inactive in the presence of As due to a significant reduction in H_2_O_2_ production, which is known to activate the NF-kB pathway (Fig. 6B). This data clearly suggests that As potentially modulates the expression of IL-8 during infection via the NF-kB pathway.

### *In vivo, C. hepaticus* levels are reduced in the liver in the presence of 0.5% As at 4 weeks post-infection

**Fig. 7:**
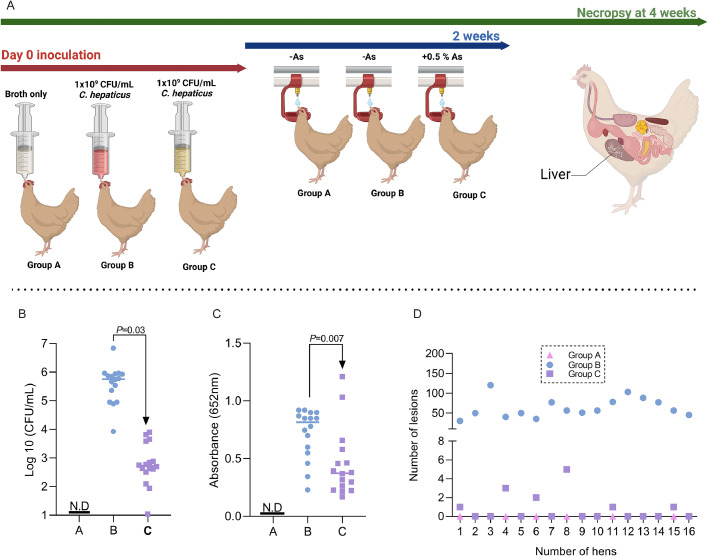
*In vivo* infection trial. Panel A – trial design (Created with Biorender.com), Panel B – quantification of *C. hepaticus* in the livers and Panel C – the levels of anti-*C. hepaticus* IgY antibody in the serum at 4 weeks postinfection. Panel D – number of liver lesions/bird. An unpaired *t*-test was used with *P* value indicated on the graph.

Quantitative PCR was used to quantify the *C. hepaticus* in liver samples (Fig. 7B). No birds from Group A were identified as positive for *C. hepaticus*. However, all the 44 birds infected in Group B, were identified as positive for *C. hepaticus* at 4 weeks post-challenge, with CFU/mL ranging from 4 × 10^4^ to 3.8 × 10^7^. In Group C, at 4 weeks post challenge, a significant decrease (*P*=0.03) in *C. hepaticus* counts in the liver was observed for all 44 birds in the presence of 0.5% As (∼ 10^3^ CFU/mL). Moreover, we show that the levels of anti-*C. hepaticus* IgY antibody in the serum is significantly (*P*=0.007) lower (Fig. 7C) in the presence of 0.5% As, suggesting that the observed effect is directly linked to the reduction in infection levels. At necropsy, we counted the number of SLD lesions on livers and observed that As significantly impacted the number of lesions identified (Fig. 7D). This small-scale experimental *in vivo* infection suggests that As can indeed reduce SLD incidence in hens at 4 weeks post-*C. hepaticus* infection. Further larger trials are required to determine if such a decrease is sustainable in the long term in industrial settings.

### The effect of As on the *in vivo* intestinal barrier

**Fig. 8:**
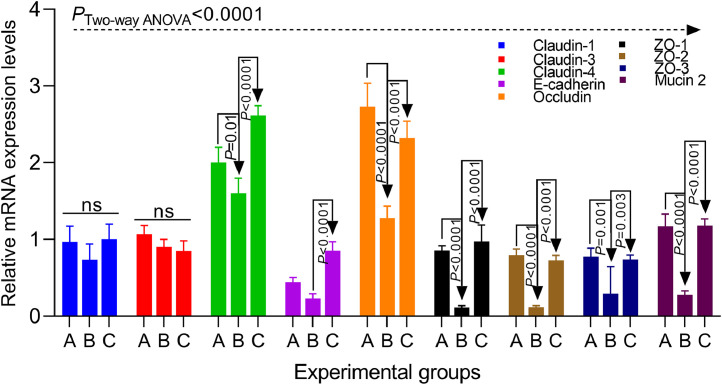
The relative mRNA expression levels of intestinal barrier-related genes. Data was analysed using the Two-way ANOVA and Bonferroni’s test for multiple comparisons to assess for significance. The *P* values are indicated on the graphs.

Based on our *in vitro* observations that As improves the transepithelial resistance (TEER) in *C. hepaticus*-infected LMH cells, we have also measured the relative mRNA expression levels of the major proteins involved in maintaining the integrity of the intestinal barrier *in vivo*, in ileal sections (Fig. 8). Except for Claudin-1 and Claudin-3 changes were observed in all other proteins investigated. In the absence of As, the infected Group B showed a significant decrease (*P*=0.01) in Claudin-4 mRNA levels compared to the uninfected control group A. However, in group C (+As), these levels were significantly increased (*P*<0.0001) compared to group B. The levels of E-cadherin (*P*<0.001), Occludin (*P*<0.0001), ZO-1 (*P*<0.0001), ZO-2 (*P*<0.0001), ZO-3 (*P*=0.003) and Mucin 2 (*P*<0.0001) were also significantly increased in the infected and As-treated group C when compared to the infected and the untreated group B. These results confirm the *in vitro* observation that As improves the epithelial barrier and negatively impacts the ability of *C. hepaticus* to infect.

## Discussion

During the last decade, researchers have explored a variety of compounds, both synthetic and natural, with potential anti-*C. hepaticus* activity. Some antimicrobial compounds, including phytochemicals (such as plant essential oils and alkaloids), organic acids, and feed nutraceuticals, have garnered interest as promising interventions, particularly for use in antibiotic-free or organic production systems (Becerra et al., 2025; Quinteros et al., 2021) .

Our results show that the antimicrobial mixture (As) was able to reduce bacterial growth up to 2% when bacterial killing was noted. Concentrations of 0.5% and 1% were identified as growth inhibitory and considered subinhibitory according to previous studies (Balta, Linton, Pinkerton, Kelly, Ward, et al., 2021; Bunduruș et al., 2023). The direct mechanism of interaction between natural antimicrobials, such as organic acids, is still unclear. However, in our study, we show that the antimicrobial mixture (As), which included organic acids, initially exhibited chemoattractant activity that immediately led to a decrease in motility. The ability of organic acids to act as a chemoattractant for *C. jejuni* was described several years ago (Hugdahl et al., 1988) and more recently, it has been identified that organic acids can act as signals to attract or repel microbes since they can act as a source of carbon or nitrogen (Bertin et al., 2003). *C. hepaticus* has an increased capacity to invade LMH cells, an immortalised chicken hepatoma cell line, and was shown to induce disease when artificially inoculated in mature layer birds (Van, Elshagmani, et al., 2017). In our study, we confirm the affinity of *C. hepaticus* for LMH cells and additionally show that the presence of As during the infection assay significantly reduces the bacterial total adhesion to the cell surface. *Campylobacter* spp. usually infects the host epithelium by disrupting cellular tight junctions (Alemka et al., 2012). We demonstrate in this study that the antimicrobial mixture effectively enhances the strength of LMH cell tight junctions, as indicated by the TEER measurements.

Following infection, IL-8 initiates the inflammatory response against *Campylobacter* spp. Infections, requiring both NF-κB and AP-1 activation for *CXCL8* gene transcription (Eucker et al., 2014). We have previously demonstrated that As can reduce *Enterocytozoon hepatopenaei* (EHP) infection in primary gut epithelial cells and inhibit the H_2_O_2_-activated NF-κB pathway (Bunduruș et al., 2023). In this study, we have identified a similar path and demonstrated that As can modulate the activation of the NF-κB pathway in infected LMH cells, directly impacting IL-8 ortholog expression. The NF-κB pathway is regulated in the presence of As through the reduction of oxidative stress (H_2_O_2_ levels) in infected cells.

Organic acids in mixtures (e.g., lactic, fumaric, citric, and sorbic) have been previously shown to possess antimicrobial properties, as well as stimulate gut health and digestibility (Dibner & Buttin, 2002). Additionally, it is noteworthy that they can enhance proteolytic activity in the gut through their overall beneficial effect on gut physiology (Adil et al., 2010). Host-derived proteases are considered to be in the first line of defence against bacterial infections, and it has been shown that they can complement the antimicrobial effect of antibiotics and eliminate bacterial pathogens (Chen et al., 2021). This beneficial effect on gut physiology can also impact proteolytic activity within the gut, increasing protease activity and potentially degrading the N-linked glycans attached to proteins. This process can be reversed by inhibiting protease activity (López-Otín & Bond, 2008). *C. hepaticus*, similarly to *C. jejuni,* share an N-glycosylation system with a role in survival and virulence in poultry (broilers or hens) (McDonald et al., 2023). Our study shows increased proteasome activation in infected LMH cells when exposed to the antimicrobial mixture (As), suggesting its ability to initiate the first line of defence against the pathogen. Indeed, these observations were translated *in vivo*, as our work clearly shows a significant decrease in liver *C. hepaticus* levels in the presence of 0.5% As in the drinking water. Anti-*C. hepaticus* antibodies are produced and rapidly detected in poultry upon *C. hepaticus* infections (Muralidharan et al., 2022). Interestingly, the levels of *C. hepaticus* anti-IgY antibodies decrease in the presence of As, indicating that they are not directly responsible for the reduced levels of infection detected but are instead a consequence of it. This result has further led to a reduction in the number of lesions detected on the liver.

## Conclusions

**Fig. 9:**
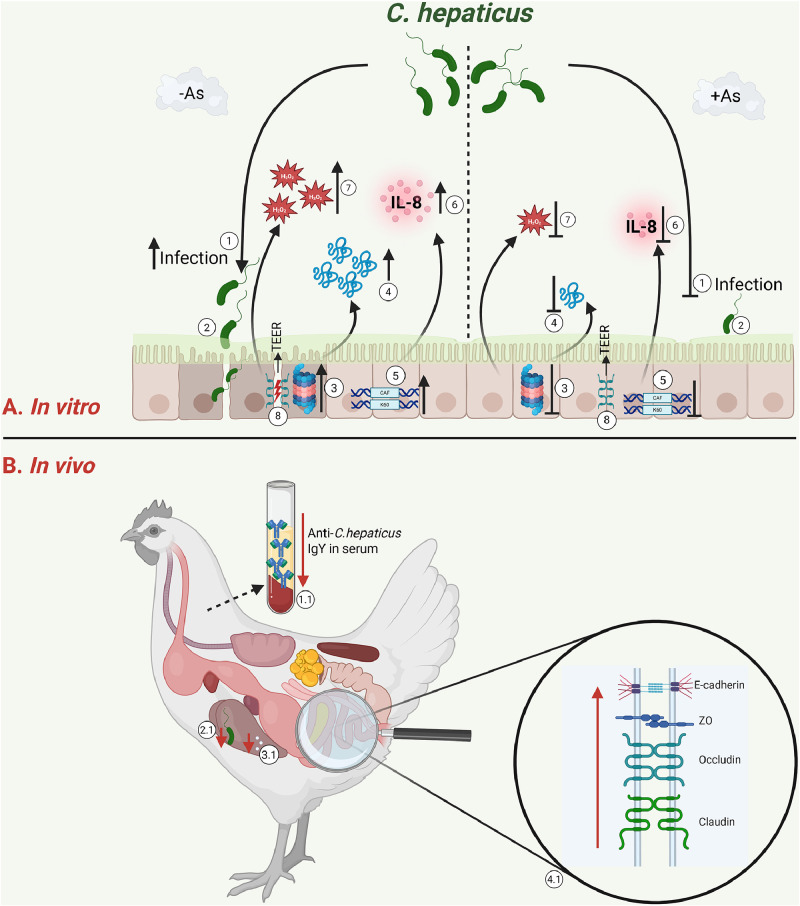
The *in vitro* and *in vivo* As mechanisms of action. A: *In vitro* - Mechanism of As impact on *C. hepaticus* infection of LMH cells. 1-2 *C. hepaticus* adherence to LMH cells; 3 – proteasome activation; 4 – protease release; 5-6 – IL-8 ortholog stimulation; 7 – H_2_O_2_ release. B: *In vivo*, As leads to reduced Anti-*C. hepaticus* antibodies in the serum (1.1), as a result of lower infection rates (2.1), a lower number of liver lesions (3.1) and a stronger intestinal barrier (4.1). Created with Biorender.com.

Our findings, summarised in Fig. 9, contribute to a growing body of evidence supporting the use of natural antimicrobials as viable alternatives to traditional antibiotics in poultry production. The modulation of immune signalling pathways, such as NF-κB, and the enhancement of host defence mechanisms like proteasome activation, underline the multifaceted benefits of As in managing *C. hepaticus* infections. Furthermore, the ability of As to reduce oxidative stress and limit bacterial invasion highlights its potential role in improving gut health and resilience in commercial poultry flocks. Our results are in accordance with this statement, as we show that the antimicrobial mixture reduces the levels of *C. hepaticus* in the liver and the number of liver lesions *in vivo*. The low levels of anti-*C. hepaticus* IgY antibodies in the serum of hens receiving As, suggest that host antibodies do not directly cause the reduction in invasion levels detected in the liver but instead involve an additional anti-pathogenic effect by directly modulating bacterial virulence factors such as motility, as observed in this study. We have also confirmed *in vivo* that As plays an essential role in maintaining the integrity of the intestinal barrier during infection. Future research should focus on the long-term impacts of As supplementation *in vivo* and its integration into broader disease management strategies for sustainable poultry farming. Collectively, these findings highlight the promising role of antimicrobial mixtures, particularly those containing organic acids, as alternative methods for controlling *C. hepaticus* infection and reducing the incidence of spotty liver disease (SLD) in poultry flocks. While the observed reductions in bacterial load and liver lesions are encouraging, it remains essential to elucidate further the mechanisms underlying these effects and to conduct more extensive, field-based studies to confirm efficacy and long-term safety. The integration of such interventions could represent a significant advancement in sustainable poultry health management, especially in contexts where antibiotic use is restricted or undesirable.

## Funding resources

This work was funded by Environtech Ireland and the University of Life Sciences King Mihai I of Romania from Timisoara doctoral funds.

## CRediT authorship contribution statement

**Ana-Maria Imbrea:** Validation, Methodology, Investigation, Formal analysis, Data curation. **Igori Balta:** Writing – original draft, Validation, Resources. **Sorin Morariu:** Project administration, Investigation, Data curation. **Lavinia Stef:** Methodology, Formal analysis. **Ioan Pet:** Project administration, Formal analysis, Data curation. **Claudia Loredana Crista:** Methodology, Investigation. **Ducu Stef:** Software, Resources, Project administration, Investigation. **Florica Morariu:** Writing – review & editing, Writing – original draft, Supervision. **Nicolae Corcionivoschi:** Writing – review & editing, Writing – original draft, Supervision, Funding acquisition.

## Disclosures

The authors declare that they have no known competing financial interests or personal relationships that could have appeared to influence the work reported in this paper.

## Data Availability

Data can be made available on request
